# Influence of Hydrogen on the Fracture Resistance of Pre-Strained Steam Generator Steel 22K

**DOI:** 10.3390/ma15196596

**Published:** 2022-09-23

**Authors:** Maciej Dutkiewicz, Oksana Hembara, Yaroslav Ivanytskyi, Mykola Hvozdiuk, Olha Chepil, Mykhailo Hrynenko, Nazar Hembara

**Affiliations:** 1 Faculty of Civil and Environmental Engineering and Architecture, Bydgoszcz University of Science and Technology, 85-796 Bydgoszcz, Poland; 2 Karpenko Physico-Mechanical Institute, National Academy of Sciences of Ukraine, 79601 Lviv, Ukraine; 3 Institute of Civil Engineering and Building Systems, Lviv Polytechnic National University, 79000 Lviv, Ukraine

**Keywords:** true stress-strain curves, fracture energy, hydrogen concentration, pre-strain, hydrogen embrittlement

## Abstract

In the paper, experimental studies of the hydrogen and pre-strained effect on fracture resistance of steam generator steel 22K were carried out. Special cylindrical samples were loaded up to fracture under a uniaxial tensile test with different pre-strained degrees and hydrogen charged times of the material. Stress-strain curves «*S_i_*–*e_i_*» were plotted. The true strain «*e_i_*» in the local volume was determined using the method of optical-digital image correlation (ODIC). The results showed that the hydrogen influence is practically absent in the elastic area of strain. The fracture energy of steel 22K decreases under the hydrogen influence and pre-strain in all investigated cases. It is shown that during six months of air exposure, with the 0% pre-strained samples release almost all hydrogen. In pre-strained samples, the hydrogen concentration decreased by 1–3% compared to the initial values. This indicates that they have trapped hydrogen that cannot escape on its own. Hydrogen embrittlement (HE) indexes for 0% pre-strained samples at different levels of hydrogen-charging calculated by the strain and energy approaches are equal to each other. There is a difference in the values of the HE index depending on the hydrogen-charge time for pre-strained samples. This indicates that both strain and strength characteristics of the material, which are integrally taken into account in the energy approach, are sensitive to HE.

## 1. Introduction

Elements and structures under operating conditions are exposed to multiple destruction processes [1]. Engineering practice constantly faces hydrogen-induced cracking (HIC) problems, hydrogen-induced brittleness, stress corrosion, and hydrogen embrittlement (HE).

HIC [2] is defined as cracking in low-to medium-strength steels where cracking is driven by the precipitation of gaseous hydrogen molecules within the crack, typically occurring in sour (H_2_S-containing) environments. It is a complicated phenomenon, encompassing a surface reaction for hydrogen uptake, hydrogen diffusion to vulnerable microstructural sites, hydrogen gas precipitation creating an incipient crack, and crack growth driven by hydrogen gas pressure within the crack. While HIC has been studied for decades, understanding of the critical factors controlling each step of the phenomenon has been elusive. The maturation of many characterization techniques gives hope that a full mechanistic understanding may occur shortly.

HE is a common, dangerous, and poorly understood cause of failure in many metal alloys. In practice, it is observed that different types of damage to industrial components have been tied to the presence and localization of hydrogen in metals [3]. This hydrogen is induced in the material during electrochemical reaction and high-pressure gaseous hydrogen environment [4].

There has been significant advancement in modeling of hydrogen-related phenomena. Most of the work [5,6,7] are focused in translating the hydrogen mechanisms and damages in constitutive equations.

There is also significant works [8,9,10,11] performed during the last 20 years using multiscale modeling schemes that attempt to tackle the underlying physics of hydrogen interaction with steels.

Hydrogen mechanisms [12] clearly show that hydrogen affects steel in the micro-nanoscale, e.g., interaction with voids, dislocations, segregates, etc. In this regard, depending on the predominant places of hydrogen accumulation in the material, one or another mechanism of its influence on the characteristics of strength, plasticity, and crack resistance of low-alloy steels is implemented, which ultimately determines their performance.

In the modern literature [2,3,4,5,6,7,8,9,10,11,12,13,14] the following main mechanisms of the influence of hydrogen on the service characteristics of structural metals and alloys are considered and discussed hydrogen-enhanced decohesion (HEDE) and hydrogen-enhanced localized plasticity (HELP). Since over a wide range of hydrogen concentrations the enhancement in the mobility of dislocations was detected from very low to high, it can be concluded that the HELP mechanism might be active, but in varying degrees, depending on the concentration of hydrogen in metal. On the other hand, the prerequisite for the activation of the HEDE mechanism is based on the hypothesis that internal hydrogen lowers the cohesive strength by dilatation of the atomic lattice, which results in a very sudden and sharp ductile-brittle transition (ductility loss), is reaching sufficiently high - so-called “critical” hydrogen concentration in a metal. These two seemingly opposing hydrogen embrittlement mechanisms (HEDE and HELP) are extremely difficult to detect simultaneously. Activation of the particular mechanism of HE and the extent of its influence on the fracture process is primarily caused by the successive processes at the micro and nano levels in the fracture process zone at the crack tip. Taketomi et al. [15] conducted their study applying the dislocation dynamics calculation, which clearly indicates that the possible change of hydrogen embrittlement mechanisms in alpha iron, from HELP to HEDE, depends on the boundary environmental and mechanical conditions: hydrogen concentration and applied stress intensity rate. Recently, simultaneous action of the HEDE and HELP mechanisms were detected, depending on the local concentration of hydrogen in investigated low-carbon steel [14]. To explain the experimentally observed dual nature of sample destruction, the idea of a “hybrid mechanism” HELP + HEDE was proposed (a review of the results can be found in [12]).

Modeling schemes performed in the continuum domain dealing with HE could be used for integrity assessment and prediction of remaining life of steel equipment, provided that databases exist on hydrogen influence on steels in various testing conditions, e.g., different hydrogen charging conditions, testing arrangements and measured parameters, correlation of measured parameters to hydrogen content in the steel, etc.

During the last decades are very limited publication [16] on the effect of cold work, e.g., pre-strain hardening. However, in engineering practice, steels often undergo plastic strain before service during the fabrication process (such as cold working), and also strain hardening is often used to improve the strength and local dent resistance. Pre-strain could affect HE susceptibility of steels. Up to now, many reports have reported that HE was increased due to pre-strain for stainless steels and high strength steels. Wang et al. [17] revealed that, when the pre-strain of 304L was higher than 10%, the pre-strain greatly enhanced HE of the steel. It was suggested that this phenomenon was mainly attributed to the strain-induced martensite transformation in 304L steel. The higher of the pre-strain, the higher content of martensite transformed, thus the increased HE occurred. Li et al. [18] found that, for high strength steels, the amount of released diffusible hydrogen was increased with a higher pre-strain. When the pre-strain was greater than 3%, the HE was dramatically increased. However, some conflicting results were also present. Mine et al. [19] reported that pre strain had little influence on hydrogen diffusivity in 316L and 310S steels, in which almost no martensite was transformed. More recently, Chen et al. [20] found improved resistance to HE of warmed 304 steel under high-pressure hydrogen atmosphere. It could be concluded from the above investigations that it is still necessary to elucidate the effect of pre-strain on the HE susceptibility in steels.

The paper investigated the effect of pre-strain on hydrogen embrittlement of steam generator steel 22K from the group of low-carbon structural alloys.

Steel is used for the production of structural parts of heat-generating equipment and pipelines, designed for long-term or constant exposure to temperatures reaching +350 °C. It is also used for the production of boiler equipment and pressure vessels in the conditions of thermal power plants and various industrial enterprises. Most often, the 22K steel alloy is used for: casing cladding of steam generators; blades and wheel rims of Francis turbines; distribution rings, welded pipes, supporting metal structures of the industry; bottoms, flanges, welded and integral drums of waste and steam boilers; combustion chambers of gas turbines.

Long-term operation of elements of structures of nuclear and thermal power plants in conditions of elevated temperatures and interactions with hydrogen-containing and corrosive environments requires researchers to use approaches that take into account changes in the physical and mechanical characteristics of metal when reassigning the period of operation or extending the service life. It was established that changes can manifest in local volumes in the form of internal and surface damage [21,22,23,24]. For a reliable assessment of the strength and deformability of metal after long-term operation, it is necessary to use criteria that would take into account changes in the characteristics of the metal in the local volume. Therefore, the use of standard methods for determining the characteristics of strength and deformability is insufficient, since the integral assessment can lead to significant errors. Standard approaches to such assessments are not entirely satisfactory, as they do not provide for control of the stress-strain state in operational conditions. The paper proposes an approach that involves using a non-contact method of control during the sample deformation in laboratory conditions and also provides an opportunity to control the most stressed areas of the structural element under operational conditions. In particular, the method of optical-digital correlation of images (ODIC) [25,26] makes it possible to control the sample deformation for a specific area and to determine the place where the stress-strain state is homogeneous from the point of view of the strain and stress distribution in the limited area. In addition, this method makes it possible to determine strain in two directions. Determination of the characteristics of strength and deformability in local volumes ensures the invariance of the established features. That is, they do not depend on the scale factor.

## 2. Material and Experimental Procedures

Determination of physical and mechanical characteristics of the studied material was carried out on the basis oт plotted complete equilibrium diagrams “*P*-Δ*l*” (*P*–load, Δ*l*–relative elongation of the sample material in the zone of maximum strain based on 1 mm). Values of true stresses *S_i_* in the zone of maximum strain were obtained by the formula: *S_i_ = P_i_/F_i_* where *F_i_*–current values of the cross-sectional sample area, including the change in its (neck) diameter. The value of the true strain e in the local volume was calculated based on the data of displacements in two directions based on 0.5 mm using an ODIC [25].

The true fracture diagram can be used to analyze the stress-strain state of engineering objects in the vicinity of stress concentrators, where stresses occur in the material that exceeds the elastic limit. In load-bearing elements of structures or machine parts, similar problems can arise during the elastoplastic strain of the material in small areas near holes, cuts, etc.

In the paper, a technique for plotting true stress-strain diagrams considering the hydrogen medium is developed based on the ODIC method, which was used to determine the true strain in a sample where the stress-strain state is homogeneous. According to this method, local strain, the place of the beginning of destruction, and its distribution over the entire surface of the sample were determined. The use of this technique significantly increases the accuracy of the research and also reduces the complexity of experimenting.

### 2.1. Material and Samples

**Figure 1:**
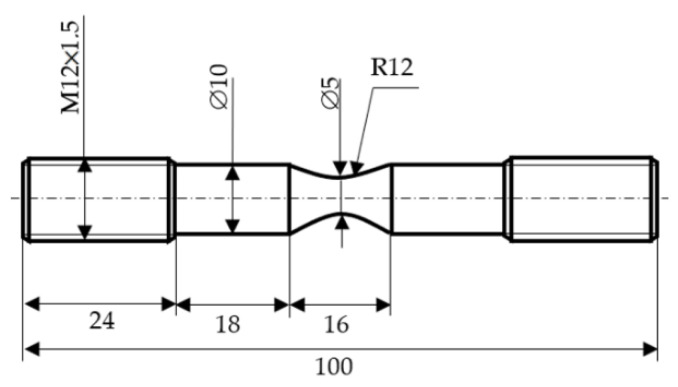
Schematic illustration of the geometry of tensile test specimens.

Modified Bridgman cylindrical steel 22k samples were made to plot fracture diagrams (Figure 1), at the quantity of 3 samples for different research parameters. Chemical composition (mass, %) of the material was: 0.17–0.24% C; 0.17–0.37% Si; 0.35–0.65% Mn; ≤0.035% P; ≤0.040% S; ≤0.25% Cr; ≤0.30% Cu; ≤0.30% Ni. Such a modified design of the Bridgman sample makes it possible to unambiguously determine the value and place of local elastic-plastic deformation, the formation of a “neck”, and fracture.

**Figure 2:**
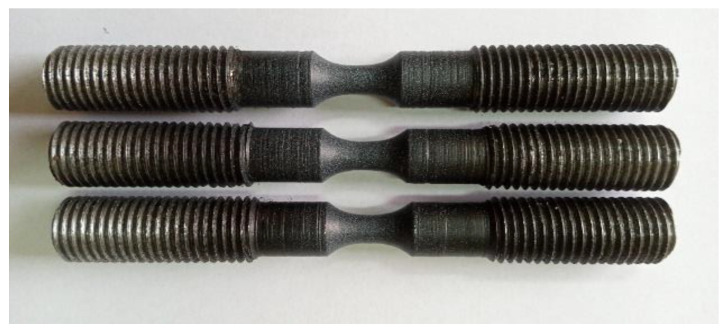
The general appearance of experimental metal specimens after drawing a dot pattern.

For the correct application of the ODIC method, a base was applied with black paint to the specially prepared working surface of the samples. Next, a stochastic dot pattern was sprayed on this base with white paint (Figure 2).

Experimental studies were carried out as follows. The sample was loaded in tension on an FP-100 electro-mechanical breaking machine, with simultaneous registration of the load force on the computer by the machine’s standard inductive dynamometer. The rate of deformation of the sample was 2.0 × 10 s^−1^.

In parallel with the load, the elongation of the central working area of the sample was recorded remotely using a digital camera to determine the amount of local deformation. The obtained experimental data were processed using Power Graph 3.3.8 software (DISoft, Moscow, Russia).

### 2.2. Preparation of Pre-Strained Samples

**Figure 3:**
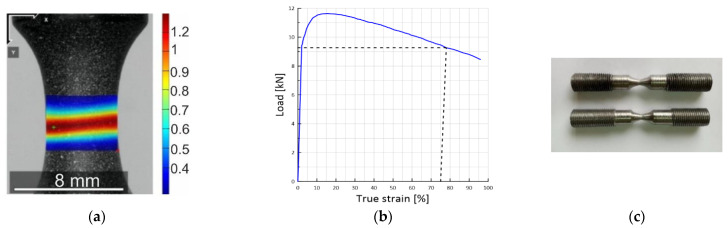
(**a**) experimental sample ODIC image, (**b**) force P versus true strain e and (**c**) general appearance of pre-strained samples.

To prepare samples with a pre-strain of 10%, 20%, 50%, and 75% strain diagrams were plotted in coordinates “*P*–*e*”, where *P*–load, [kN], *e*–true strain, [%] determined on the basis of 1 mm. experimental samples were installed in the grippers of an electro-mechanical tensile machine FP-100. Load *P* was recorded with a machine dynamometer. Measurement of local displacement of the surface in the sample neck on the basis of 1.0 mm was carried out by the ODIC method. The images of the sample for processing by the ODIC method (Figure 3a) were obtained by an optical system based on a Toupcam UCMOS 10000KPA industrial digital camera (Touptek, Hangzhou, China) with a Xenoplan lens (Schneider Optics Xenoplan, Bad Kreuznach, Germany), rigidly fixed on a special platform to the moving traverse of the machine. Camera resolution is 8 MP (3264 × 2448 pixels). During the experiment, a video was recorded with a shooting frequency of 24 fps, which was converted into separate frames. During loading, the working surface of the sample was serially photographed, and the first frame corresponds to the moment of the start of loading. Whereas the frequency of registration of efforts using the analog-to-digital converter (ADC) is 50 fps. This makes it possible with sufficient accuracy to establish the moment of fracture of the sample. Strain values were calculated using the displacement values [26].

(1) $$\varepsilon_{xx}=\frac{\partial u}{\partial x}+\frac{1}{2}\left[{\left(\frac{\partial u}{\partial x}\right)}^{2}+{\left(\frac{\partial v}{\partial x}\right)}^{2}\right],$$

(2) $$\varepsilon_{yy}=\frac{\partial v}{\partial y}+\frac{1}{2}\left[{\left(\frac{\partial u}{\partial y}\right)}^{2}+{\left(\frac{\partial v}{\partial y}\right)}^{2}\right],$$

where:

$\varepsilon_{xx}$–strain components (across the sample–perpendicular to the load);

$\varepsilon_{yy}$–strain components (along the sample, parallel to the load);

u ,v–deformation components perpendicular and parallel to the loading axis.

In total, about 5000 frames were recorded during the loading of the sample, providing the construction of a fairly smooth curve based on the results (Figure 3b). From the resulting diagram, the magnitude of the force corresponding to the given strain was set at 10, 20, 50, and 75% when passing beyond the elastic limit of the sample from the material under study. The samples prepared in this way (Figure 3c) were used to conduct experimental studies under different conditions of hydrogen saturation.

### 2.3. Hydrogen-Charge Condition

**Figure 4:**
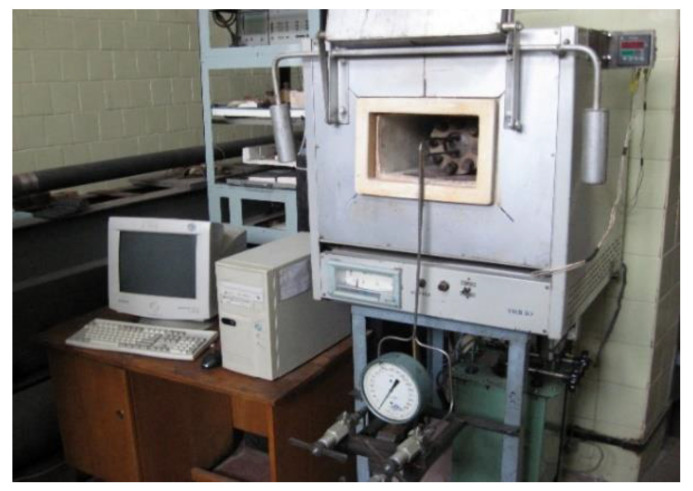
Installation for hydrogen-charging of samples.

The samples prepared for the experiment were hydrogen-charged from the gaseous phase in a special hermetic chamber (Figure 4) made of stainless steel, under controlled pressure and temperature of the environment. The hydrogen-charge temperature was 400 °C, the pressure was 6 MPa, and the holding time was: 24; 48, and 96 h.

### 2.4. Hydrogen Concentration Measurement

Hydrogen concentration was measured using a LEKO DH-603 gas analyzer (Leco Corporation, St. Joseph, MI, USA), which determines the concentration of residual hydrogen, or diffuse and residual hydrogen in metal (by using an optional glow module) in iron and iron-containing alloys. Residual hydrogen analysis is carried out by placing a pre-weighed sample in a furnace, where hydrogen is released during hot extraction in the gas stream.

The concentration of hydrogen absorbed by the material was determined for areas of the sample deformed to various degrees of damage after its testing and fracture. For this, a fragment weighing 5 ÷ 10 g. was cut out of the fractured sample in the fracture zone where the material is plastically deformed and weighed using the LECO device. In addition, a fragment was cut from this sample in the region without residual strains. The amount of absorbed hydrogen for the cut fragments was determined. The hydrogen content is measured by a thermal conductivity cell; the results are given in ppm.

### 2.5. Determining of Fracture Energy

To study the effect of pre-strain on the change in the fracture energy, as an invariant characteristic of resistance, samples were tested in their initial state. As a result of experiments in the initial state, it was established that for steel 22K, the base of 1.0 mm for determining the true strain is optimal, while the calculated strain *e_i_* is independent, and the stress-strain state is homogeneous.

The true stresses *S_i_* were determined for each stage of loading *P_i_* taking into account the change in the cross-sectional area of the sample: *F_i_*. (*S_i_ =* *P_i_/F_i_*).

Using the testing data, a true fracture diagram was plotted in coordinates: «*S_i_–e_i_*».

The fracture energy of the material is determined by the formula:

(3) $$W_{c}\left(x,y,z\right)=\int_{0}^{e_{c}}S\left(e\right)de,$$

where *S*—stress values; ε—strain values; *e_c_*—critical fracture strain.

As soon as t experiment was conducted, fragments were cut from the samples in the fracture zone and the concentration of hydrogen *C_H_* was determined using a gas analyzer LEKO DH-603.

### 2.6. Calculation of HE Index

For the quantitative description of hydrogen embrittlement of samples according to the NASA standard NASA 8–30744 [27] the HE index is used, calculated from the loss of plasticity using the strain approach,

(4) $$\omega_{\epsilon }=\left(\varepsilon_{C}-\varepsilon_{H}\right)/\varepsilon_{C},$$

where *ε_C_* and *ε_H_* are respectively the fracture strain of hydrogen-free and hydrogen-charged samples.

However, it is well known [12], that hydrogenation changes the mechanical properties of the material and, as a rule, this manifests itself in an increase in strength and a decrease in plasticity. As a result, its energy reserves change, which should lead to a change in the characteristics of fracture resistance. For an objective assessment of structural elements, it is necessary to use approaches based on the definition of invariant characteristics that do not depend on load conditions and scale factor. To solve these problems, an energy approach is proposed in the paper, which involves determining the invariant characteristics of the material strain resistance and fracture on samples in laboratory conditions. In particular, the paper introduces the HE index, calculated based on the loss of energy reserves.

(5) $$\omega_{W}=\left(W_{C}-W_{H}\right)/W_{C},$$

where *W_C_* i *W_H_*—are respectively the fracture energy of hydrogen-free and hydrogen-charged samples.

## 3. Experimental Results and Discussion

### 3.1. Stress-Strain Curves

According to the results of the research of the tested samples made of 22K steel, stress-strain curves were plotted in «*S-e*» coordinates under different conditions of pre-strain and hydrogen-charge, which were compared with the data of the tested samples in air.

**Figure 5:**
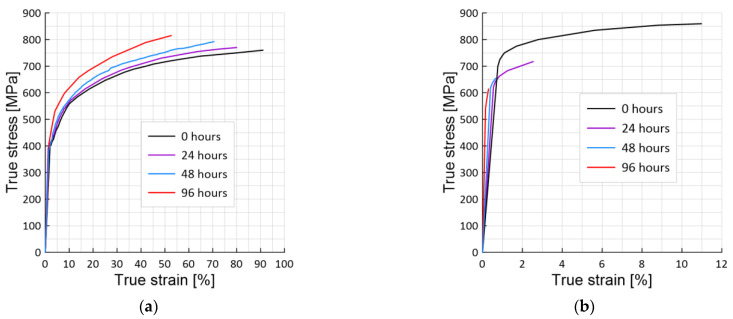
Stress-strain curves for hydrogen-charged and hydrogen-free samples with 0% (**a**) and 75% (**b**) pre-strain at ambient temperature.

Stress-strain curves (Figure 5) are presented for both hydrogen-charged and hydrogen-free samples with 0% (Figure 5a) and 75% (Figure 5b) pre-strain at ambient temperature.

For the pre-strained samples charged with hydrogen, the stress-strain curves were affected by the hydrogen for all samples at different pre-strained levels, mainly manifesting with a sizeable reduction for the fracture strain. The greater the time of exposure in hydrogen, the greater this effect.

**Figure 6:**
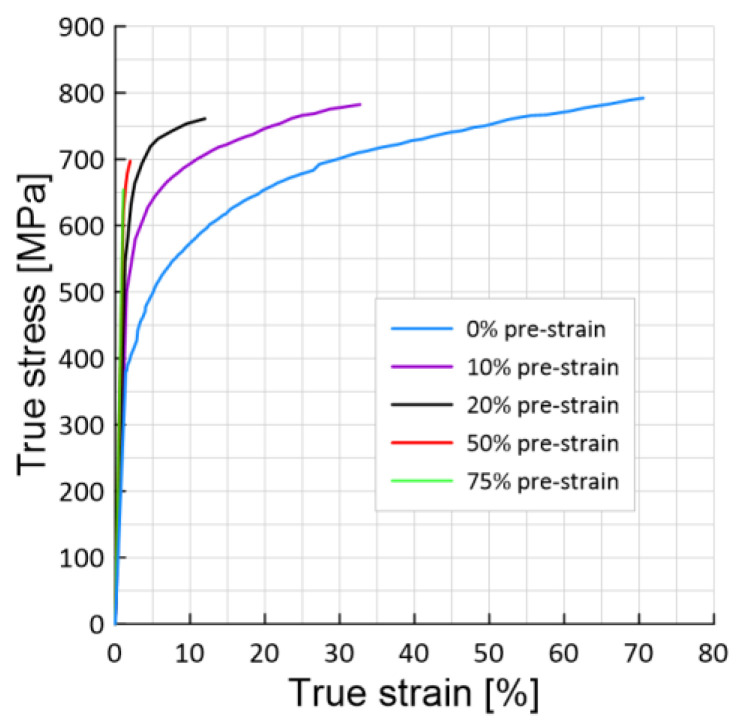
Stress-strain curves for hydrogen-charged samples for 48 h.

Stress-strain curves (Figure 6) for hydrogen-charged samples for 48 h with 0%, 10%, 20%, 50% and 75% pre-strain at ambient temperature are plotted.

The fracture stress of samples hydrogen-charged for 48 h in the initial state (Figure 6) increases by 5% compared to the hydrogen-free material (Figure 5a), and the value of the fracture strain decreases by 23%.

As can be seen from Figure 6, the maximum true fracture stress for hydrogen-charged (48 h) samples with 10% and 20% pre-strain are slightly reduced by 2% and 4%, respectively. An increase in the value of the pre-strain to 50% leads to a 12% decrease in the fracture stress. A further increase in true pre-strain reduces the failure stress to 18%. Therefore, the degree of preliminary deformation does not have a critical effect on the reduction of the true fracture stress.

Figure 6 shows that the influence of hydrogen and pre-strain was mainly manifested in a significant decrease in the fracture strain. Thus, with the previous deformation of the sample by 10%, it decreased by 54%, and by 20%–by 83%. Increasing the pre-strain to 50 and 75%, reduces the fracture strain by 96 and 98%, respectively.

Hence, for the hydrogen-charged pre-strained samples, the stress-strain curves were affected by hydrogen for all samples at different levels of pre-strain, mainly manifesting in a significant reduction in strain to failure. The longer the hydrogen-charged time, and therefore the hydrogen concentration, the greater the effect. In the elastic region, deformation under the influence of hydrogen is practically not observed.

### 3.2. Effect of Hydrogen-Charge Time and Pre-Strain on the Hydrogen Concentration

**Figure 7:**
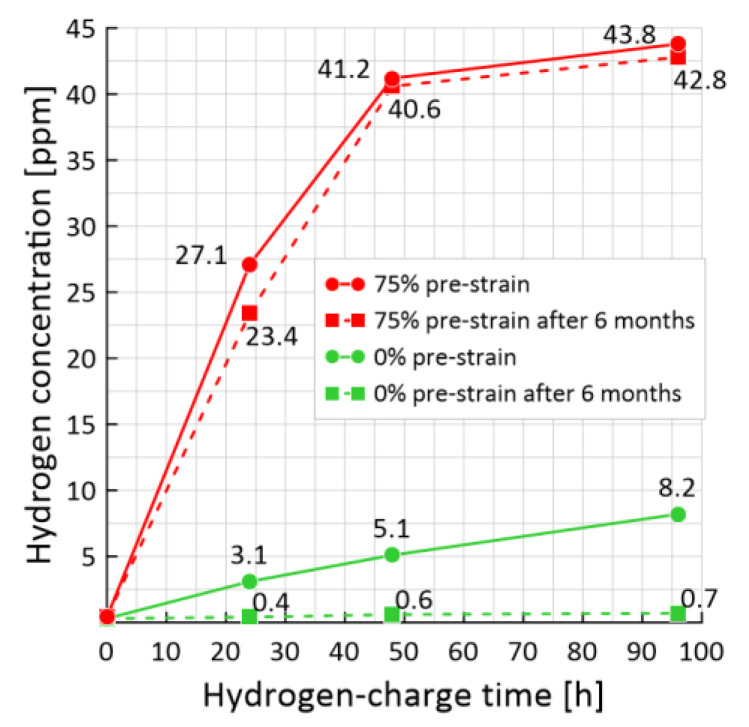
The effect of hydrogen-charged time on the hydrogen concentration measured immediately after the experiment (solid curves) and after 6 months of sample storage (dashed curves): red curves—with 75% pre-strain, green—with 0% pre-strain.

The results obtained in the paper make it possible to analyze the change in hydrogen concentration depending on the pre-strain levels and after a certain aging time. Figure 7 shows the hydrogen concentration on the hydrogen-charging time. The results of the presence of residual hydrogen under different conditions of preliminary hydrogen-charge are presented.

For the 0% pre-strained material, an almost linear dependence is observed, and the hydrogen concentration increases to 8.2 ppm during the hydrogen-charging time of 96 h (green solid curve, Figure 7). After keeping the samples in the air for 6 months, it was established that there is practically no residual hydrogen in them (green dashed curve, Figure 7). This indicates that the 0% pre-strained samples contained diffusible hydrogen, which escaped over time.

In pre-strained samples up to 75%, the hydrogen concentration increases linearly upon reaching saturation. The value of the concentration in the case of saturation for 96 h increases to 43.8 ppm, i.e., it increases almost 9 times compared to the strain-free case (Figure 7, red solid curve). After exposure for 6 months in air in these samples, the value of hydrogen concentration practically did not change (red dashed curve, Figure 7).

**Figure 8:**
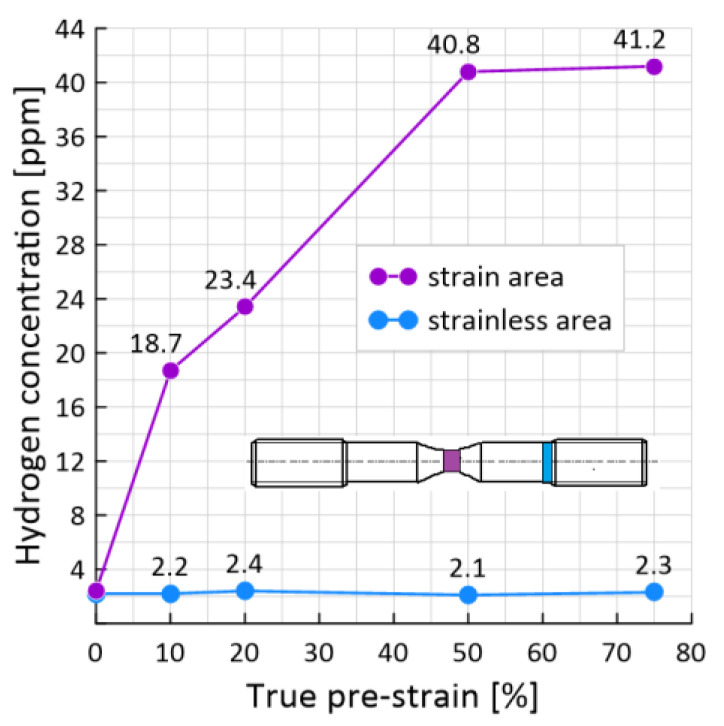
The effect of pre-strain on the hydrogen concentration measured immediately after the experiment in different parts of the sample.

Figure 8 shows a comparison of the hydrogen concentration in the un-deformed part of the sample and in the neck for hydrogen-charged samples for 48 h with 0%, 10%, 20%, 50% and 75% pre-strain.

In the un-deformed part of the sample, we observe almost the same amount of hydrogen concentration. In the neck of the sample, the concentration of hydrogen increases with the level of preliminary deformation. If in the 0% pre-deformed sample the concentration was 2.4 ppm, then in the 10% pre-deformed sample the concentration was already 18.7 ppm (an increase of 7%). A further increase in the level of pre-deformation of the samples to 20% and 50% leads to an increase in the concentration to 23.4 ppm and 40.8 ppm, respectively.

This indicates that under conditions of plastic strain, hydrogen is located in internal cavities from which it cannot degas even at ambient temperature. This damage process is not reversible, for hydrogen degassing from steel, additional energy is needed to remove it from the cavities formed in the material.

This is due to various mechanisms of hydrogen influence [28]. In previously undeformed samples, the BO mechanism is implemented, which consists in the diffusion of atomic hydrogen into the zone of increased stresses in the metal lattice. In pre-deformed samples, the NIS mechanism is implemented, which occurs in carbon or low-alloy steels when atomic hydrogen diffuses into them and forms molecular hydrogen. The formation of molecular hydrogen is facilitated by internal trap defects that appear as a result of previous deformation. The formation of molecular hydrogen creates internal pressure on the material and initiates cracking. The coexistence of different mechanisms of hydrogen exposure and their simultaneous action in steels is still not well understood, while the recognition of the dominant mechanism is an extremely difficult and critical task.

### 3.3. The Effect of Hydrogen-Charge Time on the Local Fracture Strain

**Figure 9:**
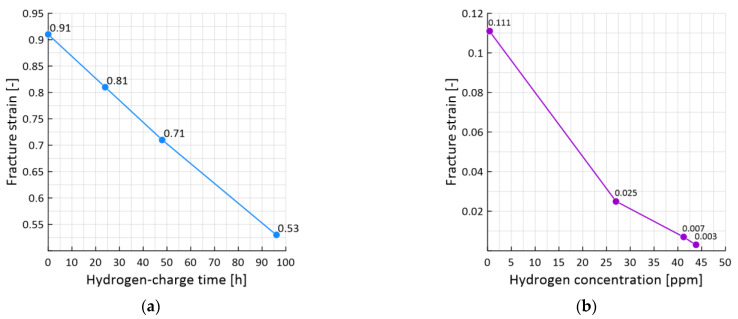
The effect of hydrogen-charge time on the fracture strain: with 0% (**a**) and 75% (**b**) pre-strain samples.

Hydrogen affected the stress-strain curves for all samples at different levels of pre-strain and different hydrogen-charged times, in particular, manifesting itself in a significant reduction of the local fracture strain. A comparison of the local strain to failure for the hydrogen-free and hydrogen-charged samples with 0% (a) and 75% (b) pre-strain is presented in Figure 9. The fracture strain of sample with 0% pre-strain (a) was reduced from 0.91 to 0.8 after hydrogen charging during 24 h (a reduction of 12%), to 0.71 after hydrogen charging during 48 h (a reduction of 22%), to 0.53 after hydrogen charging during 96 h (a reduction of 42%).

However, the stronger effect of hydrogen was manifested in samples with 75% pre-strain (b). The local fracture strain of samples with 75% pre-strain was reduced from 0.11 to 0.03 after hydrogen charging during 24 h (a reduction of 72%), to 0.007 after hydrogen charging during 48 h (a reduction of 94%), to 0.003 after hydrogen charging during 96 h (a reduction of 97%).

The significant influence of the pre-strain of the samples on the fracture resistance of both hydrogen-free and hydrogen-charged samples is worth noting. Thus, the fracture strain for the hydrogen-free samples decreased from 0.91 for 0% pre-strain sample (a) to 0.11 for 75% pre-strain sample (b) (a reduction of 88%). Similar reductions were also found in hydrogen-charged samples.

For samples with a different degree of pre-strained, a proportional dependence is observed. So, for example, for the 10 and 20% of pre-strained, during the hydrogen-charge time of 24 h the fracture strain is reduced by approximately 5 and 15%. For 48 h, by 25 and 35%, and during the hydrogen-charge of 96 h, by 44 and 55%. A sharp jump in the reduction of deformation is observed for 50% of the pre-strained of the sample. During the hydrogen-charge time of 96 h, it decreases by 90%.

So, as shown by the results of studies of samples with of 75% pre-strained, their resistance to destruction sharply decreases. As a result of the pre-strain, defects were formed in the material of the sample that retain hydrogen, there is practically no plastic strain, and the material fractures after reaching the elastic limit.

### 3.4. The Effect of Hydrogen-Charge Time on the Fracture Energy

The fracture energy (3) was calculated using the next formula, replacing the integration with the summation of elementary areas,

(6) $$W=\frac{1}{2}\overset{i*}{\underset{i=0}{\sum }}\left(S_{i+1}-S_{i}\right)\left(e_{i+1}-e_{i}\right)$$

where i*—element number (or time step) in which the strain energy reaches its maximum value,

$S_{i}$—the value of the true stresses in the *i*-th element,

$e_{i}$—the value of the true deformations in the *i*-th element.

**Figure 10:**
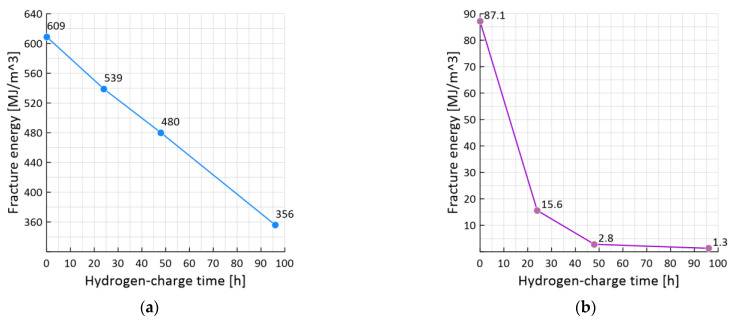
The effect of hydrogen-charge time on the fracture energy: with 0% (**a**) and 75% (**b**) pre-strain samples.

Using the plotted true stress-strain fracture diagrams (Figure 5 and Figure 6), the fracture energy *W_C_* and *W_H_* was calculated for both hydrogen-free and hydrogen-charged samples, respectively. Figure 10 shows the results of the hydrogen-charge time effect and, accordingly, the hydrogen concentration on the fracture energy for 0% pre-strained (Figure 10a) and pre-strained up to 75% samples (Figure 10b). The value of the fracture energy of steel 22K in the initial state is equal to 609 MJ/m^3^. The true fracture stress is 740 MPa, and the fracture strain is 0.91. With an increase in the hydrogen-charging time, the value of the fracture energy reaches a minimum during a hydrogen-charging after 96 h and is equal to 356 MJ/m^3^, i.e., it decreases by 42%. While the value of true fracture stresses increases by 9.2%. At the same time, the value of true strain also decreases by 42%. The result indicates an inversion of the fracture stresses *S_i_* and the true critical strains *e_i_* for steel 22K. In this case, the evaluation of the stress-strain state by one parameter is not a sufficient condition.

The fracture energy is reduced by 7 times in pre-strained samples with large pre-deformations 75%, compared to the pre-undeformed sample (from 609 MJ/m^3^ to 87 MJ/m^3^). As the hydrogen-charge time increases, the value of the fracture energy reaches a minimum during hydrogen-charging after 96 h and is equal to 1.3 MJ/m^3^, i.e., it decreases by 99%, while the fracture strain decreases by 97%. In pre-strained samples with large pre-deformations 75% there is practically no plastic component of deformation. The metal under such hydrogen-charge conditions breaks brittle without signs of plastic strain. In this state, the material has internal defects and the highest concentration of absorbed hydrogen.

For samples with a different degree of pre-strain, namely 10%, 20% and 50%, a decrease in the value of the fracture energy is also observed due to an increase in the time of hydrogen-charging, and therefore from the concentration of hydrogen.

The magnitude of the fracture energy is sensitive to changes in the resistance of 22K steel to deformation under the action of hydrogen and the level of the pre-strain. For samples without pre-strain, hydrogen reduced the fracture energy by about 50% compared for the hydrogen-free samples.

### 3.5. HE Index

**Figure 11:**
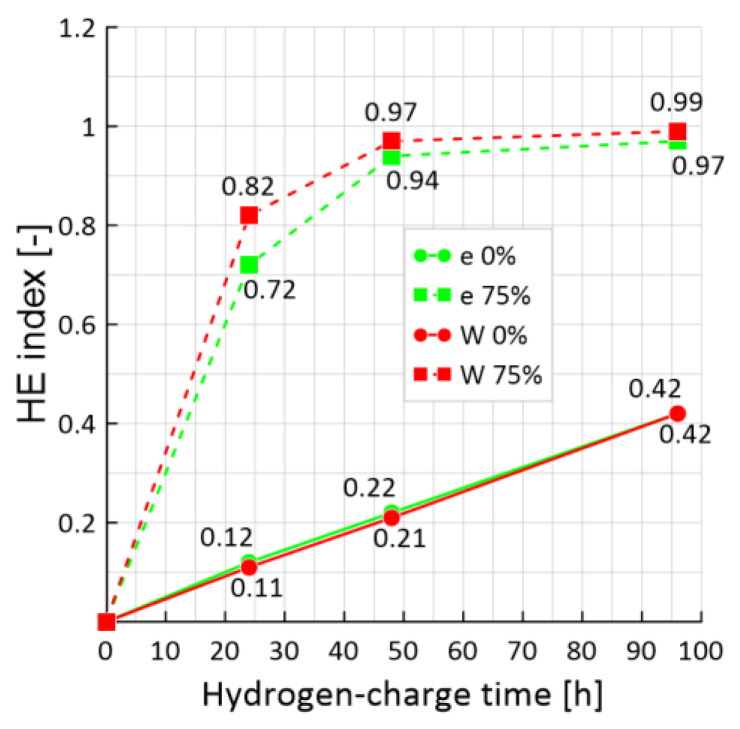
HE index: with 0% (solid curves) and 75% (dashed curves) pre-strain samples; red curves—according to the energy approach, green curves—according to the strain approach.

For the quantitative analysis of hydrogen embrittlement of the samples, the HE index was used, calculated from the plasticity loss (4) and the energy reserves loss (5). Figure 11 shows that for the 0% pre-strained sample, the HE index increases gradually with increasing hydrogen-charge time. It should be noted that the 0% pre-strained sample already showed a fairly significant loss of plasticity after hydrogen-charging (up to 42%). However, the largest loss is observed in 75% of pre-strained samples. Moreover, hydrogen-charging within 24 h has already caused a rapid increase in the HE index to 0.72 according to the strain approach (4) and 0.82 according to the energy approach (5). A further increase in hydrogen-charge time led to an increase in the HE index.

Moreover, the indices of HE for 0% pre-strained samples at different levels of hydrogen-charging calculated by Formulas (4) and (5) are equal to each other. However, already for 75% of pre-strained samples, there is a difference in the values of the HE index up to 12% for samples with hydrogen-charging within 24 h and 2–3% for 48 and 96 h of hydrogen-charging.

This suggests that both strain and strength characteristics of the material are sensitive to HE. Therefore, it is more appropriate to use such an integral characteristic as the energy of elastoplastic strain, which takes into account the change in both stresses and strains due to the action of external factors.

## 4. Conclusions

Most studies on the effect of hydrogen on the resistance to destruction of structural elements are carried out on the basis of the hydrogen embrittlement index, which is evaluated by the change in the elongation of the original and specially prepared sample. The paper proposes to estimate the hydrogen embrittlement index based on the energy approach, which is based on the determination of the destruction energy in the local volume. The ODIC method is used to determine local deformations.

The main conclusions of the paper can be summarized as follows:

- Almost no hydrogen effect is observed in the elastic region of strain.
- The exposure time of the samples in gaseous hydrogen in a special hermetic chamber affects the level of metal hydrogen-charge of the samples in different ways. In 0% pre-strained samples, an almost linear dependence of hydrogen concentration on the hydrogen-charge time was established. While in pre-strained up to 75% samples, the concentration increases rapidly after 48 h of hydrogen-charging and then changes slightly.
- The effect of further air exposure of pre-hydrogen-charged samples on the concentration of hydrogen in them was established. It was shown that almost all hydrogen was released from 0% pre-strained samples during 6 months of air exposure. A completely different picture is observed in samples pre-strained to 75%. In the samples the hydrogen concentration decreased by 1–3% compared to the initial values. This indicates that trapped hydrogen remained in samples pre-strained up to 75%, which cannot escape on its own.
- The significant influence of the pre-strain of the samples on the fracture resistance of both hydrogen-free and hydrogen-charged samples is worth noting. Thus, the fracture strain for the hydrogen-free samples decreased from 0.91 for 0% pre-strain sample to 0.11 for 75% pre-strain sample (a reduction of 88%). Similar reductions were also found in hydrogen-charged samples.
- With 75% pre-strain, the fracture energy decreases by 7 times compared to the 0% pre-strained sample from 609 MJ/m^3^ to 87 MJ/m^3^. As the hydrogen-charge time increases, the value of the fracture energy reaches a minimum during hydrogen-charging after 96 h and is equal to 1.3 MJ/m^3^, i.e., it decreases by 99%.
- HE indices for 0% pre-deformed samples at different levels of hydrogen-charging calculated by the strain and energy approaches are equal to each other. In addition, already for 75% of pre-strained samples, there is a difference in the values of the HE index up to 12% for samples with hydrogen-charging within 24 h and 2–3% for 48 and 96 h of hydrogen-charging. This suggests that both the strain and strength characteristics of the material are sensitive to HE, which are integrally taken into account in the energy approach.
- The simultaneous action of both HE mechanisms (HELP+HEDE) is responsible for reducing the plasticity of steel. Although a critical hydrogen concentration was not determined in this study, the results clearly indicate that the degree of reduction in fracture strain and fracture energy values can be successfully correlated with the hydrogen concentration in the metal. The fracture energy of steel decreases with increasing hydrogen-charge time, and therefore hydrogen concentration, regardless of which type of embrittlement mechanism is dominant.

## Data Availability

Not applicable.
